# The impact of bone morphology on the outcome of the pivot shift test: a cohort study

**DOI:** 10.1186/s12891-017-1798-4

**Published:** 2017-11-17

**Authors:** Thomas Branch, Shaun Stinton, Adrija Sharma, Frederic Lavoie, Christian Guier, Philippe Neyret

**Affiliations:** 1 University Orthopedics, Decatur, GA USA; 2 ArthroMetrix, 441 Armour Place NE, Atlanta, GA 30324 USA; 3 Center for Musculoskeletal Research, MABE Department, University of Tennessee, Knoxville, TN USA; 4 Department of Orthopedic Surgery, Hôpital Notre-Dame, CHUM, Montréal, QC, Canada; 5 San Francisco Sports Medicine and Orthopaedic Surgery, San Francisco, CA USA; 6 Department of Orthopaedic Surgery, Centre Albert-Trillat, Hôpital de la Croix-Rousse, Lyon, France

**Keywords:** Pivot shift, ACL, ACL reconstruction, Automated clinical knee examination, Knee laxity

## Abstract

**Background:**

The presence of a positive pivot shift after surgical repair of the ACL is considered an important indicator of a failed reconstruction. The ability to predict the result of a pivot shift test after an ACL reconstruction using variables that can be measured prior to surgery could provide an indication of which patients may be at-risk of a poor surgical outcome.The purpose of this study was to determine whether structural characteristics of the femur and tibia, measured using plain radiographs, were associated with the result of the pivot shift test in unilateral ACL reconstructed patients.

**Methods:**

Sixteen patients who had undergone unilateral ACL reconstruction were divided into two groups based on the results of manual pivot shift testing: 1) Pivot group; and 2) No pivot group. All patients had standing true lateral radiographs of both knees. Structural measurements of the tibia and femur were made on both knees. In addition, two new variables were created to describe the tibiofemoral mismatch: 1) Femur Tibia Size Ratio (FTSR); and 2) Tibia to Posterior Femoral Condyle Ratio (TPFCR). These measures were compared within groups and between groups.

**Results:**

None of the individual structural characteristics were significantly different when compared between groups. No individual structural characteristics had a significant association with the presence of a positive pivot shift. When a between-group analysis was performed, both the FTSR (*p* < 0.03) and the TPFCR (*p* < 0.01) were significantly different between the Pivot group and the No Pivot group. A larger FTSR ratio, or a larger femur relative to the tibia, was associated with a positive pivot shift. A smaller TPFCR ratio, or a smaller tibial depth relative to the depth of the lateral posterior femoral condyle, was associated with a positive pivot shift.

**Conclusions:**

Structural characteristics in the lateral femoral condyle and lateral tibial plateau were found to be associated with the presence of a positive pivot shift. These characteristics could separate between patients in the Pivot group and the No Pivot group. Two indices, the FTSR and the TPFCR, provided better predictive value than individual characteristics in identifying patients with a knee that was structurally “at-risk” for developing a positive pivot shift.

## Background

The pivot shift test is highly specific for diagnosis of anterior cruciate ligament (ACL) laxity. Clinical use of this test has increased in popularity since a relationship between the pivot shift and patient satisfaction was reported by Kocher et al. [1, 2]. The pivot shift is a complex maneuver in which a supine patient’s knee is initially allowed to sag into full extension while an examiner applies an internal rotation force and a valgus force causing the tibia to sublux anteriorly. The examiner then flexes the tibia while continuing to apply the internal rotation force and valgus force. A positive pivot shift is indicated when the tibia’s position on the femur suddenly reduces anteriorly into its anatomical position during flexion of the tibia. This subluxation of the tibia on the femur generally occurs at approximately 20–30° of knee flexion [3].

The presence of a positive pivot shift after surgical repair of the ACL is considered an important indicator of a failed reconstruction [4]. The ability to predict the result of a pivot shift test after an ACL reconstruction using variables that can be measured prior to surgery could provide an indication of which patients may be at-risk of a poor surgical outcome. This additional knowledge may affect surgical decision making (i.e. a surgeon may consider alternative treatments such as additional lateral reinforcement or the use of a double-bundle ACL reconstruction technique in at-risk patients).

Structural characteristics of the femur and tibia have been previously investigated to determine their ability to predict the result of the pivot shift test. Structural characteristics may be associated with the pivot shift and may predispose an ACL reconstruction to failure [5–7]. Structural characteristics such as tibial plateau convexity [8], femoral notch width [9], tibial slope [10–12], and the relative size of the tibia and femur [6] have been investigated to determine their relationship to knee instability and the presence of a positive pivot shift. While these previous studies investigated individual structural measures and their association with the result of the pivot shift test, it is possible a linear combination or ratio of multiple structural characteristics may better describe the tibiofemoral mismatch.

The purpose of this study was to determine whether structural characteristics of the femur and tibia, measured using plain radiographs, were associated with the result of the pivot shift test in a population of unilateral ACL reconstructed patients. It was hypothesized that the result of the pivot shift test would be associated with structural characteristics of the femur and tibia. It was further hypothesized that a structural index combining multiple structural characteristics would be more specific than any individual characteristic in predicting the presence of a positive pivot shift after ACL reconstruction.

## Methods

Sixteen patients who had undergone unilateral bone-patellar tendon-bone ACL reconstruction and who were available for followup testing (not deceased and still living in the area) were retrospectively reviewed an average of 9 years after surgery (range: 8 to 10 years). Subjects consented to participate in the study. Institutional review board approval was not required for this study at the institution where testing was performed (Centre Albert-Trillat, Hôpital de la Croix-Rousse, Lyon, France) at the time of the study. All surgeries were performed by a single author between January 1998 and May 1999. Eleven males and 5 females were studied. Demographic information is shown in Table 1.

**Table 1:** Patient Demographics

	Median Value (Range)
Age (years)	40 (26–48)
Height (m)	1.7 (1.6–1.8)
Weight (kg)	70 (55–95)
Reconstructed knee extension (degrees)	0 (0–3)
Reconstructed knee flexion (degrees)	150 (135–160)

The ACL reconstruction technique used the middle third of the patellar tendon with one bone block press fit in the femur and another bone block fixed in the tibia with an interference screw. Extra-articular reconstruction (EAR), when performed, consisted of a gracilis tendon autograft routed through a hole in the femoral bone block, with both limbs passed under the lateral collateral ligament and attached via bone tunnels on either side of Gerdy’s tubercule with the knee in neutral rotation and flexed to 30° [13].

At the time of review, each patient completed three validated subjective questionnaires: the Knee Injury and Osteoarthritis Outcome Score (KOOS), the International Knee Documentation Committee subjective score (IKDC), and a modified Visual Analog Scale (VAS). Each patient underwent physical examination by two independent orthopaedists who did not perform the surgeries. Each physician performed manual knee laxity tests (Lachman-Trillat and Pivot Shift) and instrumented knee laxity tests (KT-1000 performed at 67 N, 89 N, 133 N and manual maximum force) on the reconstructed knees [14]. No laxity tests were performed on the healthy knees. The manual maximum force was the force applied during the manual maximum tibial displacement test. All laxity tests were performed in a blinded (i.e. the examiner was not aware of the injury type) and randomized fashion (i.e. the order of the examiners was randomized).

**Fig. 1:**
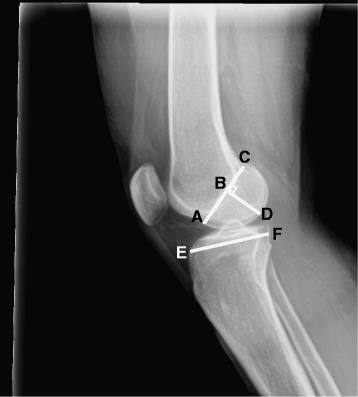
Example measurements of bony anatomy in the knee. The measurements taken were the AP depth of the anterior femoral condyle at Blumensaat’s line (**a**-**b**), the depth of the posterior lateral femoral condyle at Blumenstaat’s line (**b-c**), femoral condyle length from Blumensaat’s line to the most distal portion of the condyle (**b**-**d**), and the AP depth of the tibia (**e**-**f**)

All patients had a standing true lateral radiograph of the reconstructed knee taken at nine year followup. These radiographs were measured for AP tibial depth, depth of the anterior lateral femoral condyle at Blumenstaat’s line, depth of the posterior lateral femoral condyle at Blumenstaat’s line, and femoral condyle length defined as the perpendicular distance from Blumenstaat’s line to the most distal part of the femoral condyle (Fig. 1). From these measures of the bony morphology of the knee, two new variables were created to describe the tibiofemoral mismatch: 1) Femur Tibia Size Ratio (FTSR); and 2) Tibia to Posterior Femoral Condyle Ratio (TPFCR). The FTSR was calculated using a composite size of the lateral femoral condyle achieved by adding all structural condylar measurements (depth of the anterior lateral femoral condyle, depth of the posterior lateral femoral condyle, and femoral condyle length) and dividing by the AP depth of the tibia. The TPFCR was calculated using the AP depth of the tibia divided by the depth of the posterior lateral femoral condyle.

### Statistical analysis

Using the results of the manual pivot shift test on the reconstructed knee, two groups were created based on the presence or absence of a positive pivot shift (Group 1: Pivot group and Group 2: No Pivot group). Structural measurements of the tibia and femur were made on both the healthy knee and the reconstructed knee. To compare knees within each group (left versus right or reconstructed versus healthy), paired comparisons were made using Wilcoxon signed rank test. To compare means between patients who had a positive pivot shift on their reconstructed knee and those who did not, the Wilcoxon rank sum test was used. To compare differences in between-group proprotions, the Fisher’s exact test was used. A *p*-value of <0.05 was considered statistically significant.

## Results

There were 10 patients in the No Pivot group and 6 in the Pivot group. There were no statistically significant differences between the groups in pre-operative laxity, the development of meniscal lesions, or degenerative changes during the follow-up period. There were nine medial meniscectomies, six in the No Pivot group and three in the Pivot group. There were 4 lateral meniscectomies, 4 in the No Pivot group and none in the Pivot group. Three patients had OA in at least one compartment: three in the No Pivot group and none in the Pivot group. There were 5 females and 1 male in the Pivot group and 10 males and no females in the No Pivot group. There were 3 patients with EAR and 7 patients without EAR augmentation in the No Pivot group and 2 patients with EAR and 4 patients without EAR in the Pivot group.

### Individual structural characteristics of the distal femur and proximal tibia

The median values and ranges of structural characteristics are shown in Table 2. The characteristics are also broken down by group (Pivot group and No Pivot group). None of the individual structural characteristics were significantly different when compared between groups. There were no significant differences found for any of the characteristics between the right and left knees or between the reconstructed and healthy knees within each group. No individual structural characteristics had a significant association with the presence of a positive pivot shift.

**Table 2:** Structural characteristics of the distal femur and proximal tibia in the reconstructed knees

Indices	Overall median	Overall range	Pivot group median	Pivot group range	No pivot group median	No pivot group range	*p*-value*
AP Tibial Depth (mm)	64	50–72	61	50–70	67.5	59–72	0.111
Anterior Femoral Condyle Depth (mm)	33.5	27–38	31.5	27–34	33.5	28–38	0.345
Posterior Femoral Condyle Depth (mm)	24	19–28	24.5	19–28	24	21–26	0.738
Femoral Condylar Length (mm)	32	25–38	31.5	25–38	32	29–35	0.855
Femur Tibia Size Ratio (FTSR)	1.40	1.2–1.5	1.43	1.4–1.5	1.36	1.2–1.4	0.007
Tibia to Posterior Femoral Condyle Ratio (TPFCR)	2.62	2.3–3.1	2.53	2.3–2.6	2.74	2.5–3.1	0.007

*2-sided *p*-value Wilcoxon rank sum test

### Constructed indices: FTSR and TPFCR

**Fig. 2:**
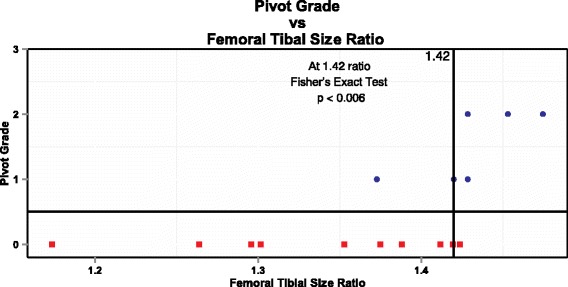
The Femur Tibia Size Ratio versus Pivot Shift Grade in the reconstructed knees. A ratio of 1.42 or higher is associated with a positive pivot shift. Squares represent patients in the No Pivot group and circles represent patients in the Pivot group

**Fig. 3:**
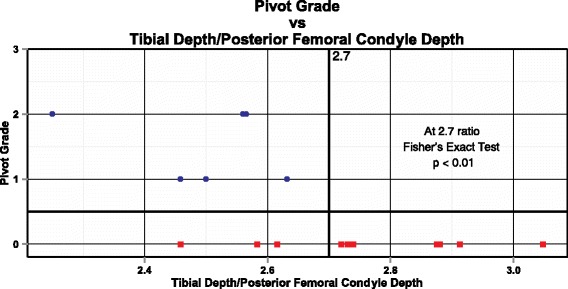
The Tibia to Posterior Femoral Condyle Ratio vs Pivot Shift Grade in the reconstructed knees. A ratio under 2.7 is associated with a positive pivot shift. Squares represent patients in the No Pivot group and circles represent patients in the Pivot group

When a between-group analysis was performed, both the FTSR (*p* < 0.03) and the TPFCR (*p* < 0.01) were significantly different between the Pivot group and the No Pivot group. A larger FTSR ratio, or a larger femur relative to the tibia, was associated with a positive pivot shift. A smaller TPFCR ratio, or a smaller tibial depth relative to the depth of the lateral posterior femoral condyle, was associated with a positive pivot shift. Graphically, the distribution of each group for FTSR is shown in Fig. 2 and the distribution of each group for TPFCR is shown in Fig. 3. There were no significant differences found for these indices between the right and left knees or between the reconstructed and healthy knees within each group. In Figs. 2 and 3, the critical value for FTSR (1.42) and TPFCR (2.7) to separate between the Pivot group and the No Pivot group were picked through visual inspection. A more rigorous statistical method which uses a linear combination of all the structural characteritics to classify patients into the two groups, called Fisher’s linear discrimination analysis, is described in the Appendix.

## Discussion

The most important finding in this study is that there were unique structural characteristics of the lateral femoral condyle and the lateral tibial plateau that were associated with the result of the pivot shift test after single-bundle ACL reconstruction. These characteristics, that can be measured prior to ACL reconstruction, were able to distinguish between patients in the Pivot group and patients in the No Pivot group. The fact that a patient’s structural anatomy may have an influence on the presence of a positive pivot shift suggests that structural vulnerability that can be measured prior to surgery may be an indication of a knee that is at-risk of suffering from a positive pivot shift after reconstruction.

FTSR and TPFCR are measures specific to a single knee that do not rely on a side-to-side comparison between knees. These indices may help to identify patients whose “margin of error” for ligament reconstruction may be markedly smaller than the average knee. As shown in Fig. 3, one-third of patients with an adverse TPFCR ratio did not have a pivot shift present after ACL reconstruction. Therefore, restoration of knee stability can be achieved despite the structurally “at-risk” knee.

Losee in his 1983 article on pivot shift concepts discusses the idea of a “misfit” between the lateral femoral condyle and the lateral tibial plateau contributing to the pivot shift [15]. In a study by Bull and Amis, it was proposed that the shape of the lateral femoral condyle, the lateral tibial plateau and the slope of the plateau might correlate with the presence of a pivot shift; however, it did not [16]. In the current study, the relative shape comparison between the distal femur and proximal tibia was associated with the presence of a pivot shift. A higher FTSR ratio indicates a larger distal femur compared to the proximal tibia in the AP dimension. This may mean that a larger distal femur allows for “normal” ligament length with the smaller proximal tibia allowing for more AP excursion. Certainly, the size of the posterior lateral femoral condyle determines the length of the lateral and anterolateral ligamentous structures such that unimpeded flexion is allowed. Perhaps patients with a larger FTSR or smaller TPFCR simply have more play in the knee. Structural differences among patients, both ligamentous and bony, combine to define the individual that is at-risk for experiencing a positive pivot shift after an ACL injury and reconstruction.

The effect of bony morphology on the magnitude of the pivot shift has been reported in a study using magnetic resonance imaging to measure femoral condyle size and tibial plateau size in patients with an ACL injury [17]. Correlations between these measures of morphology and the grade of pivot shift were determined. A smaller lateral tibial plateau diameter in the medial-lateral direction may correlate with a higher grade of pivot shift. This was shown to be true for females, but not males. No significant differences were found in the other bony dimensions that were measured (AP and ML diameters of the medial femoral condyle, lateral femoral condyle, and medial tibial plateau and the AP diameter of the lateral tibial plateau). The results from the current study were similar to the study by Musahl et al. in that a smaller lateral tibial plateau indicated a trend towards having a positive pivot shift. As suggested by Musahl, it would be logical to assume that the AP width of the femur and tibia would have a greater influence on the pivot shift outcome rather than the ML width. However, in the study by Musahl et al., a correlation between ML diameter of the tibial plateau and pivot shift grade was shown, but no correlation was found with the AP diameter. The current study did find a correlation using the AP diameter as a portion of a combined indice describing the bony anatomy. The Musahl paper investigated a different patient population (grade 1 pivot shift vs. grade 2 pivot shift) than the current study which compared reconstructed knees without a pivot and reconstructed knees with a residual pivot shift. The current study also uses multiple AP measurements to create a ratio which may be more sensitive to pick up differences between groups. The current study also includes the femoral condyle length which was not measured in the Musahl paper. Ratios that included multiple structural characteristics proved to be more specific than individual characteristics in separating patients in the Pivot group and No Pivot group.

Five out of six patients who had a positive Pivot Shift in this study were female. It has been reported previously that females have more Total Leg Rotation than males [18]. By combining the increased Total Leg Rotation seen in females with the structural differences as described by the FTSR and the TPFCR, females would have a higher percentage of their population in the biomechanically “at risk” group. It follows that this “at risk” group could continue to have a positive Pivot Shift after an anterior cruciate injury and subsequent reconstruction. This is consistent with previous findings of more females than males with persistent positive Pivot Shifts after reconstruction [19]. When comparing results between females and males, females had a significantly higher FTSR (1.42 vs. 1.34; *p* = 0.04) and a significantly lower TPFCR (2.48 vs. 2.74; *p* = 0.01). Both of those trends were associated with the prescence of a pivot shift in the current study.

The additional EAR procedure did not appear to have an effect on the presence of a residual pivot shift. There were 2 patients with EAR in the Pivot group and 3 patients with EAR in the No Pivot group. When comparing all patients with an EAR to all patients without EAR, there were no statistically significant differences in FTSR (1.39 vs. 1.37; *p* = 0.76) and TPFCR (2.58 vs. 2.67; *p* = 0.78).

When comparing the three patients with OA to the patients without OA, both FTSR (1.36 vs. 1.38; p = 0.78) and TPFCR (2.79 vs. 2.63; *p* = 0.37) were statistically equivalent. All three patients with OA were in the No Pivot group. Two patients had grade 2 OA in the medial compartment with one of those patients also having grade 2 patellofemoral OA. The other patient had grade 4 OA in the medial compartment.

There are some limitations in this study. While the data collection was meticulous, the sample size is small. To verify the results of this study, a larger number of subjects would need to be examined.

## Conclusions

Structural characteristics in the lateral femoral condyle and the lateral tibial plateau were found to be associated with the presence of a positive pivot shift. These characteristics could separate between patients in the Pivot group and the No Pivot group. Two indices, the Femur Tibia Size Ratio (FTSR) and the Tibia to Posterior Femoral Condyle Ratio (TPFCR), provided better predictive value than individual characteristics in identifying patients with a knee that was structurally “at-risk” for developing a positive pivot shift.

## Figures and Tables

**Fig. 4:**
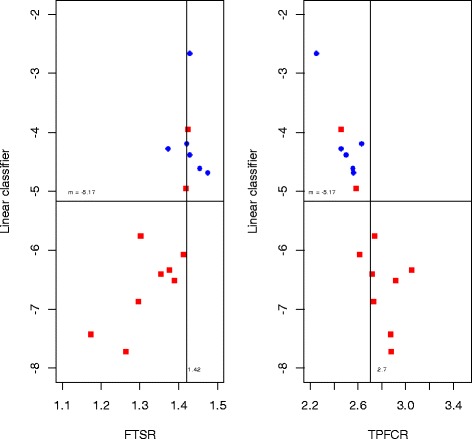
FTSR and TPFCR

## Appendix

### “Appendix – Linear Discrimination”

This appendix describes a more rigorous statistical method which uses a linear combination of all the structural characteritics to classify patients into the two groups, called Fisher’s linear discrimination analysis.

Linear discrimination is a statistical data reduction technique that looks for linear combinations of **p** variables which optimally separate objects/subjects into two (or more) groups. This is achieved geometrically by projecting the original data onto a new space in the direction where the data points are maximally separated into the two groups.

Using Fisher’s discriminant function to derive the linear classification rule, one assumes that covariance matrices between the two groups is equal, Σ_1_ = Σ_2_ = Σ. The goal is to identify the linear combination that achieves maximal seperation between the two groups. We can estimate Σ with **S**_p_, the pooled sample covariance matrix and μ_i_ with the i^th^ class sample mean vector for the **p** variables, ${\underline {\overline{x}}}_i$. It be shown that $y={\left({\underline {\overline{x}}}_1-{\underline {\overline{x}}}_2\right)}^T{S}_p^{-1}\underline{x}={l}^T\underline{x}$ maximizes the ratio of the between group variance divided by the within group variance, $$ \frac{{\left({l}^T{\underline {\overline{x}}}_1-{l}^T{\underline {\overline{x}}}_2\right)}^{\begin{array}{l}2\\ {}\end{array}}}{l^T{S}_pl} $$ . ***l*** can be shown to be the linear combination (direction) to project the data that maximally separates the data into two groups. ***l*** is unique only up to a constant so different software will scale or normalize the vector ***l***. When the assumption of equal covariance matrices is not reasonable, a quadratic discriminant analysis can be applied to relax the assumption.

For the data in the current study, the assumption of equal covariance matrices did not appear valid. For this reason, the misclassification rates of both a linear and quadratic classification rule were compared. The misclassification rates were virtually identical for these approaches so the linear results are described here. Using Fisher’s linear discrimination techniques with the the 16 injured legs, the estimated coefficients (*l*) for each of the structural characteristics were −0.0566 (anterior lateral femoral condyle), 0.5049 (posterior lateral femoral condyle), 0.1897 (femoral condyle length), −0.3400 (AP depth of the tibia). The the estimated cutoff value $m=0.5\left({l}^T{\underline {\overline{x}}}_1+{l}^T{\underline {\overline{x}}}_2\right)=-5.1664$, allocates a subject to positive pivot shift group if ${l}^T\underline {x_0}\ge m$ and to the no pivot shift group otherwise. This classification rule resulted in 8/10 patients being correctly identified as no pivot shift and 6/6 subjects as positive pivot.

The graphs below compare the results of the estimated linear classifier to each of the ratios defined in the paper. The FTSR ratio (≥ 1.42) correctly classifies 9/10 of the no pivot shift subjects and 5/6 of the positive pivot shift subjects. The TPFCR correctly classifies 7/10 of the no pivot shift subjects and 6/6 of the positive pivot shift subjects.

## Abbreviations

ACL: Anterior cruciate ligament
AP: Anteroposterior
EAR: Extra-articular reconstruction
FTSR: Femur to tibia size ratio
ML: Mediolateral
OA: Osteoarthritis
TPFCR: Tibia to posterior femoral condyle ratio
